# Lessons in learning: An effective process for engaging 12- to 14-year-olds in stem cell biology

**DOI:** 10.15252/embr.201439398

**Published:** 2014-12-17

**Authors:** Emma Kemp, Ian Chambers

**Affiliations:** 1 MRC Centre for Regenerative Medicine, University of EdinburghEdinburgh, UK; 2 Institute for Stem Cell Research, School of Biological Sciences, University of EdinburghEdinburgh, UK

Scientists wanting to reach beyond academia to improve public appreciation and understanding of research face a number of challenges. Perhaps the first of these is the question of which of the many potential audiences to address. Many scientists and science communicators focus their efforts on school children, perhaps because they are perceived to be particularly open to new ideas and knowledge. However, not all school audiences are the same. We chose to discuss concepts of stem cell science with high school students before they made the curriculum choices that put them on a path towards or away from science. Although high school outreach offers the chance to enthuse future scientists, our motivation was to reach those who would not choose science as their career. Engaging these people is essential to enhance the general level of scientific literacy in society. However, this particular audience poses some challenges, particularly as by this age, some students may already have dismissed science as irrelevant to them, while others who might be interested have little understanding of cell biology. Our challenge was therefore to convey the key concepts of stem cell biology in a way that efficiently engaged the students, demonstrated the relevance of science to their lives, was fun for all involved and enabled all students to place science appropriately within their imagined futures.

We developed a series of three lessons for 12-to 14-year-olds to address the basic concepts, applications and ethics of stem cell biology (Table1; Box 1). Working together—one of us is a scientist and the other is a science communicator—we focused on designing a set of educational modules with an emphasis on interactivity, experiments, games and discussion to actively engage students in the topic. In addition, we pursued a longer-term outreach strategy to disseminate the information and material as widely as possible internationally.

**Table 1:** Content of each of the three lessons for 12-to 14-year-olds

Lesson Title	1. Discover Stem Cells	2. CSI: Cell Science Investigators	3. Stem Cell Treatments and Ethics
Aim	To answer the question: ‘What are stem cells?’	To know what stem cells can and may be used for and to introduce the scientific method	To support discussion of ethical questions on the application of stem cells for new treatments
Learning Objectives	Know that a stem cell is a cell that can both self-renew and differentiate Know that there are different types of stem cells and be aware of where they are found Know why stem cells are important in the body	Know how stem cells may be useful in medicine and research Know an example of a disease that stem cell research may one day help Know why controls are needed to make a scientific experiment a fair test	Discuss societal/ethical considerations relating to new stem cell therapies Enable pupils to express and explain their opinions and consider other people's opinions about use of new and experimental therapies Develop discussion and scientific literacy skills
Interactive Elements	Stem cell decision-makers Blood stem cell movie Cell families card game	Make a nerve activity Use real images to identify what goes wrong with cells in MS Drug testing experiment	General dilemma and decision discussion activity Stem cell ethics scenario and opinion activity
Link	http://bit.ly/1svcTNY	http://bit.ly/1iWtJEy	http://bit.ly/QLWXKQ

**Box 1: Description of the content of the three lessons**

Each lesson uses a combination of interactive modules and facilitator-led discussion.

**Figure 1:**
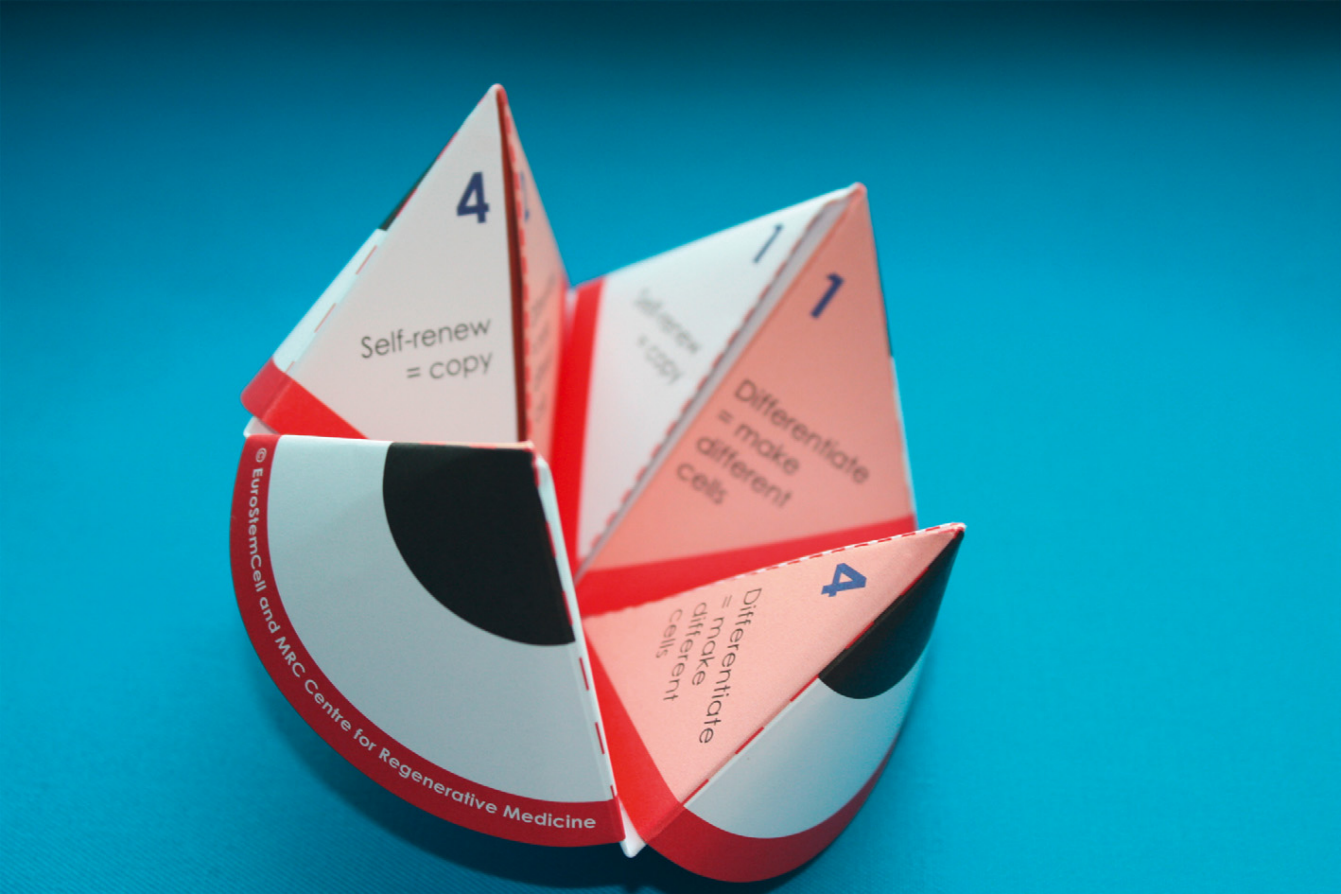
The stem cell decision-maker enables pupils to discover the properties of stem cells through a group game

The first lesson, *Discover stem cells* (http://bit.ly/1svcTNY), aims to answer the question, “What are stem cells?” It begins with an interactive module, a *Stem cell decisions* game, in which students work in small groups to discover the two defining properties of a stem cell: self-renewal and differentiation. Using a “stem cell decision-maker”, modelled on the classic folded-paper fortune teller (Fig1), students determine the fate of their equally allocated quota of blood stem cells and, in the face of stochastically drawn choices between self-renewal and differentiation, compete to maintain their pool of stem cells. The outcomes of the game are discussed as a class to reinforce understanding of what a stem cell is. Facilitators then develop this concept further by leading students through the second interactive module: a counting competition demonstrating how stem cells maintain our blood system throughout our lives. A movie of dividing cells acts as a visual summary of the concepts of renewal and differentiation. The third interactive module, the *Cell families* card game, introduces the different types of stem cells in our bodies and demonstrates the concept of multipotency. It also introduces a wild card that does not fit the same pattern as the rest of the pack: embryonic stem cells. Facilitators use a poster showing the human body and the tissues included in the card game to provide a structure for summarising the take-home messages from the game, with the embryonic stem cell wild card drawing out the concept of pluripotency and linking to the next lesson.

The second lesson, *CSI: Cell Science Investigators* (http://bit.ly/1iWtJEy), focuses on possible uses of stem cells in research and introduces the scientific method by using multiple sclerosis (MS) as an example. It begins with a quick recap activity in which students build a simple diagram summarising the two key characteristics of a stem cell. The first 10-minute interactive module introduces MS in two steps. First, the role of nerve cells is demonstrated using a line of students passing a written message hand-to-hand against the clock and acted on by the final receiver. Second, students examine real cell images in groups to determine which cells are affected by MS. Facilitators then introduce how stem cell research might contribute to the development of new treatments. The final activity is a hands-on drug testing experiment in which students determine which of several solutions has activity in a test assay. This, like most of the other modules in the lessons, can readily be adapted in length and difficulty. In this case, the task can either demonstrate the concept of a controlled experiment in simple terms, or can allow students to plan their experiment in writing and think more deeply about the importance of controls in the scientific method. The lesson plenary provides an opportunity to discuss the status of current research on stem cell therapies.

The third lesson, *Stem cell treatments and ethics (*http://bit.ly/QLWXKQ), focuses on ethical considerations of the application of stem cell research. The lesson begins with an activity entitled *Dilemmas and decisions*, in which everyday scenarios that draw directly on ethical dilemmas that 13-year-olds might face are used to set the scene. Students are asked to discuss the scenarios and think explicitly about the skills they need as a group to have a constructive debate and to reach a consensus. The following two activity modules in the lesson then introduce two different hypothetical scenarios related to stem cell treatments. Students work in groups to consider the viewpoints of a number of fictional characters and to develop and discuss their own views. A discussion at the end of the lesson compares the conclusions of different groups and produces a list of things the class believes should be considered when deciding whether a therapy should be given to patients.

The idea of engaging early-stage high school students came from a throwaway remark that Ian made during a seminar at the University of Manchester, in which he recalled a memorable open day visit he had made to the university with his school when he was 13. During that childhood visit, he had been thrilled to see a complex chemical mixture separated into its constituents a few minutes after injection into a gas chromatograph; this experience, along with lessons from an inspiring high school chemistry teacher, marked the point at which he first became drawn to science. A few weeks after the seminar, the Head Teacher of The Derby School—Ian's old high school—invited Ian to give a talk to her students about stem cell science. In preparing for the visit, Ian teamed up with Emma to maximise the potential benefit of the visit. This in turn started the development of the three stem cell lessons that are the subject of this article.

As a science communicator for EuroStemCell, Emma was aware that educational material for 12-to 14-year-olds had not begun to focus seriously on the core scientific concepts involved in stem cell research. She also knew that many stem cell researchers were enthusiastic about outreach, but were hesitant to try educating school children due to a lack of time to prepare lessons and/or a lack of confidence in their ability to engage younger students. The visit to The Derby School and the subsequent project represented an opportunity to address this resource gap and provide support to researchers wanting to engage high school students.

…our motivation was to reach those who would not choose science as their career. Engaging these people is essential to enhance the general level of scientific literacy in society

The last piece of the puzzle was the demand for such lessons. In England and Scotland, stem cell science has recently been added to school curricula (http://tinyurl.com/qbg5clt; http://tinyurl.com/n4covw4), and the topic is new and potentially daunting for many teachers. In discussion with teachers, it became clear that engaging, scientifically accurate, clear and easy-to-use lessons would meet an educational need, not only in cell biology, but also in the area of the scientific process and the role of science in society.

Our initial engagement with The Derby School eventually culminated in three lessons about the basics of stem cell biology, applications for regenerative medicine/biotechnology and the ethical implications of developing stem cell treatments (Table1; Box 1). Each lesson covers a one-hour school period with approximately 50 minutes of learning, and each can be used individually. The lessons all share a core structure consisting of three 10-to 15-minute interactive modules connected by five-minute facilitator-led sections and book-ended by 5–10 minutes at the start and end of the lesson for introductions and settling down, and for summarising key concepts and collecting feedback. Facilitators use PowerPoint slides, posters or film to support discussion and reflection on group interactive activities. We optimised this format through extensive piloting, both by ourselves and by colleagues delivering lessons at remote sites. Each round of piloting informed further development and fine-tuning of the content and materials. This iterative, collaborative development process was a key to ensuring the effectiveness of the final lessons and allowed us to identify a Goldilocks principle for achieving the right balance between delivering didactic information and enabling learning through interaction, discussion and, most importantly, fun. The process relied on a three-way collaboration between scientist, science communicator and teacher. As we progressed through the lessons and the formula for success became clearer, we were able to design and optimise materials with fewer rounds of discussion and piloting.

An important advantage of this modular approach is that the individual segments can be readily substituted to increase flexibility. These lessons can thus meet the needs of researchers visiting schools in different contexts, or demands from teachers for interactive stem cell teaching aids. Activity-centred modules supported by simple graphics-based literature also constitute a format that is easily translated and updated. Our modules have, to date, been used by more than 240 colleagues throughout Europe and beyond.

In developing our school lessons, we found that setting curriculum-relevant goals is a critical factor in obtaining support from teaching staff. To prepare for our first visit to The Derby School, we asked the host teacher what the curriculum objectives were in the early years of high school in England. We then cross-referenced our list of stem cell biology essentials with these educational priorities to create a programme that would give students a full and curriculum-relevant picture of the field.

Upon arriving in school, we quickly discovered that our aims were too ambitious and eventually unrealistic and unachievable. The teacher and, to a certain extent, the curriculum had placed particular emphasis on the need for ethical discussion. We felt strongly that such a discussion must be based on a solid understanding of the science if it is to be meaningful. As a result, we tried to achieve too much in one lesson. An introduction to the concept of a stem cell, the potential applications of stem cell biology and the ethical issues it raises might have been possible had we simply delivered a talk from the front of the class. However, students’ interest and enthusiasm must be engaged for effective learning and this would not have allowed enough time to deliver all our intended messages *and* to give students the time they needed to think, discuss, and to become actively involved in tasks.

Our modules have, to date, been used by more than 240 colleagues throughout Europe and beyond

We also underestimated some of the practical constraints of the school day. It takes time to get students settled in a classroom, while the bell at the end of the lesson is inflexible. The day of the week and time of day can affect both energy levels and how promptly lessons begin. In particular, Monday mornings tend to throw up unexpected issues such as staff absences. Concentration levels also dip after lunchtime and towards the end of the week. For these reasons, we subsequently determined that a Tuesday morning is often the best time to visit a school. At this point, the first readjustments after the weekend's freedoms have been made, but neither post-lunch drowsiness nor end-of-week restlessness has set in.

The dual challenges of defining curriculum-relevant, scientifically informed goals and making these goals realistic and achievable within the practical constraints of the school day make successful lesson design a nontrivial task. We needed to break down our original one-lesson workshop into a series of three closely related but independent one-hour lessons that would each contain an achievable set of learning objectives and, together, would convey our initially identified content. To do this, we initiated a partnership with a local teacher and evolved a collaborative process that took us from initial concepts through iterative piloting and redevelopment to dissemination of fully tested lesson materials (Table2).

**Table 2:** Several important points emerged while developing the lessons

Recommendations for developing a schools programme	
Pay attention to the curriculum and set clear learning objectives	Lessons must be curriculum relevant and objectives clear for researchers and teachers to use them and for learning outcomes to be achieved
Balance didactic and interactive activities	To maximise impact, embed plenty of fun by balancing didactic material with activities and discussion
Use a modular format for flexibility and ease of translation	A modular format enables each lesson to be tailored to the abilities of the pupils in the room and the time available and can be more readily translated, updated and replicated
Collaborate to benefit from science and communication expertise	A synergy between scientist and science communicator contributes positively to outcomes on several levels
Build a relationship with a teacher/teachers	Identify a supportive Head of School Science to allow access to classes and encourage input from teachers over an extended time and repeated visits
Embed evaluation	Plan your evaluation strategy as you plan your lesson because obtaining feedback after leaving school is extremely difficult (or impossible)
Piloting by creators AND by others is essential	Use ongoing interactive development by reiterative delivery and remote testing by colleagues to maximise value and uptake of materials
Pilot lesson guidance as well as content	Lesson guidance should balance detail with brevity to enable intuitive and creative delivery by future facilitators
Use two facilitators to deliver the lesson	A team of two facilitators improves overall delivery since change of voice and style is valuable for maintaining interest, while a dynamic interplay conveys enthusiasm and helps facilitators maintain concentration
Timing is important	Both the day of the week and the time of day affect energy levels and ease of engagement; plan a break between lessons during pilot school visits

Our first step was always to define a core message for the lesson, broken down into short, achievable learning objectives. With these objectives agreed, we brainstormed ideas for a narrative and for interactive activities. The choice of examples and activities proved easier in some cases than others. We settled fairly quickly on the blood system as our example for Lesson 1, since blood cells are amongst those most likely to be familiar to 12-to 14-year-olds, and the role of stem cells in the body can be readily illustrated by considering the lifespan of a red blood cell and the number needed by the body throughout life. However, the choice of a disease to illustrate the application of stem cell research in Lesson 2 required considerably more thought. We wanted a disease that represents an active area of stem cell research, which has a relatively straightforward underlying cellular problem and that could be easily explained to illustrate the role of stem cells in understanding disease or developing drugs. It also needed to be relevant to 12-to 14-year-olds. Diabetes came first to mind, but the likelihood that a pupil might suffer from diabetes is not low and this could put an individual in an emotionally challenging situation. We eventually chose multiple sclerosis (MS) because although it might be the case that a member of a student's family is affected, 12-year-olds themselves will not have been diagnosed with MS. Moreover, unlike other diseases including Parkinson's disease, MS is not as closely identifiable as a disease of the aged, making it easier for pupils to relate to the impact of MS. We also had the advantage that several research groups near Ian's laboratory work on MS, enabling us to obtain cell images for use in the lesson.

After our first brainstorm, Emma developed prototype slides and activity resources, which was followed by further rounds of discussion to review and improve on the materials and to iron out any problems. These revisions helped us focus on the key concepts and reduce confusion or unnecessary complexity. This approach ensured that we had a clear understanding of each other's intentions and the same expectations, which was essential to pilot the lessons together and quickly adapt to circumstances in the classroom.

It takes time to get students settled in a classroom, while the bell at the end of the lesson is inflexible

We generally scheduled three or four pilot lessons on the same day to maximise the value of each visit, and we quickly realised that a break between lessons was important to give us breathing space to adjust our plan based on experience from the previous class. Each lesson contained its own creative and conceptual challenges, some of which only became apparent during delivery. We often found ourselves making on-the-spot changes, removing or adding explanation, changing who took responsibility for introducing a module or the time for a task. Most researchers and many science communicators working in University posts are rarely, if ever, in high school for a whole day. Good facilitation demands energy and breaks are essential in maintaining energy levels. We had a great deal of fun—a valuable outcome in itself—but the unaccustomed intensity of interaction in the classroom is certainly tiring and facilitation becomes less effective if exhaustion sets in. Involving two facilitators in running the lesson helps maintain energy levels. It also hugely improves overall delivery of the lesson; change of voice and style is valuable for maintaining interest, while a dynamic interplay conveys enthusiasm and helps facilitators maintain concentration.

**Figure 2:**
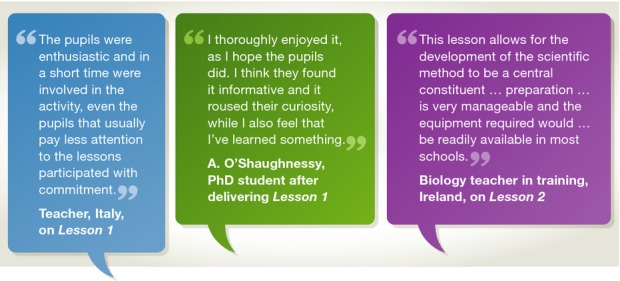
Selected feedback on the lessons

Following lesson delivery, it is vital to obtain immediate feedback from students and teachers to determine the level of success (Fig2); asking for comments after leaving the school is almost never fruitful. We created lesson-specific feedback forms with both qualitative questions about pupils’ experience of the lesson, and multiple-choice questions to see whether we had succeeded in teaching the key concepts in the lesson's objectives. In addition, we had asked our contact teacher in advance to help us gain feedback from her colleagues. Although a teacher was always present in the classroom with us and was asked for immediate feedback, time for discussion was often limited before the start of the next lesson. It was therefore also helpful to arrange a short conversation in the staff room before we left for the day to pick up on any problems and get suggestions for improvement. We took all these views back with us, added our own reflections and discussed improvements.

This process was repeated until all feedback indicated that the lesson was working well, that is, the students were engaged at—or ideally above—their normal levels; the learning objectives had been achieved by all or the vast majority of students; the materials could readily be delivered by two facilitators in the time available, allowing ample time for discussion. To establish this third point, we asked colleagues to deliver the lesson without direct coaching from us, but rather by providing the materials and a set of instructions intended to accompany online dissemination of the final completed lessons. Emma observed the first pilots by colleagues and got their feedback on what had been difficult or easy, and what was clear or unclear in the instructions. The same student and teacher feedback mechanisms as in our own pilot lessons were also applied. Our extensive pilots relied on the support of teachers, who often tell us that a packed curriculum and examination schedule leaves them with little flexibility for additional learning experiences, such as visits from researchers, unless these contribute to the curriculum. Still, most teachers would gladly welcome such opportunities to improve their students’ learning experience. Direct engagement with scientists can also be valuable to teachers themselves, since it gives them the opportunity to learn about the state of research in their field. In reality, we met teachers who had differing expectations of our visit, ranging from a keen interest in biology and enthusiasm for widening students’ experience to, in rare cases, an expectation that our visit would tick off another item on their curriculum. It was therefore important to obtain feedback on our lessons from all teachers whose classes we visited to get an unbiased picture of their effectiveness.

…most teachers would gladly welcome such opportunities to improve their students’ learning experience

Given that teachers must take time away from regular lesson plans to support a pilot lesson and participate in feedback discussions during their free time, it is no small thing to ask for access to pupils and detailed criticism. In addition, many teachers are not available for telephone calls most of the day. For an individual school visit, these challenges can be overcome by some sensible steps (Table3). For our long-term project, we were lucky to work with a very supportive school Head of Biology, who allowed repeated access to the same students and new classes over 3 years and who encouraged the other teachers to offer us the feedback and input we needed. Of course, one school does not provide an adequate picture of how the lessons will work in the full range of school environments, and so a further advantage of asking colleagues to carry out remote tests of our lessons was that we were able to gain feedback from many different schools. By the end of the development process, the lessons had been delivered to approximately 700 students by a total of six researchers.

**Table 3:** Advice for setting up a school visit

Speak with the teacher who will be your contact on the day	Do not rely solely on email to set up your visit; a phone conversation will avoid differing expectations on the day
Be informed about the curriculum	Identify the curriculum documents that apply to the school you wish to visit and ensure you have a clear understanding of their requirements
Explain concisely what you have to offer	Expect your contact teacher to be limited for time to speak on the phone; be clear and concise on what you wish to do and what it offers pupils
Be clear about what you need to deliver your activity	Tell the teacher about any specific requirements in advance, such as extra space, laboratory equipment, an AV system with sound, internet connection
Find out about the pupils	Ensure you know the class size and age before your visit; ask about ability levels and any topics you will be discussing that may be sensitive for some pupils
Do not commit to more than you can do well	Maximise value for everyone by delivering several lessons, but remember you will need breaks
Consider the school timetable	Be willing to adapt your plans to avoid unnecessary and time-consuming rearrangements of the school day; agree an arrival time that gives you time to set up
Be flexible	You may need to adjust your plans after your first conversation with the teacher
Ensure a teacher will be present	A teacher should always be in the classroom with you
Ask for feedback	Arrange in advance to collect comments from teachers and act on this feedback
Provide teachers with useful resources	You can add value to your visit by providing teachers with copies of materials they can use themselves

Once development and piloting of the lessons was completed, it was essential to ensure they could and would be used. An important factor in ensuring that others can easily use the materials is keeping text on resources to a minimum and using visuals wherever possible. Good graphics, diagrams or even video instructions make it easier for both facilitators and students to quickly grasp the purpose of an activity. Reducing both the amount and the complexity of the text also makes it easier to translate it into other languages and to disseminate it. Such considerations early in the development process ensure that all the effort invested is not lost once the lesson is completed. Although the lessons were designed for delivery by researchers, we also wanted to encourage use by teachers themselves to maximise the value of the resources. Of course, teachers’ needs and skills differ from those of researchers, and some adaptation is necessary to achieve good take-up of the lessons by both groups. Teachers will often adapt material to suit their own teaching style or the specific needs of their students. Wider dissemination to teachers should take this into account by using a format that allows editing. The lesson plan and further guidance should be kept short and to the point. This allows teachers to quickly gauge how the materials can best be used, making it more likely that they will incorporate some of the activities into their teaching.

An important factor in ensuring that others can easily use the materials is keeping text on resources to a minimum and using visuals wherever possible

An additional motivation for expanding the level of public engagement for some researchers will likely be the increasing requirement from research funders for scientists to disseminate the results of their research beyond academic journals and to foster engagement and discussion with the public (http://tinyurl.com/ljsuwz6; http://tinyurl.com/m3t37j7). Many scientists take on this challenge with enthusiasm and energy; some with great skill. However, many others will just employ a science communicator to carry out this onerous task with instructions and guidance from the scientist. Our collaboration was therefore unusual—being an equal partnership with a long-term commitment by each of us—and initiated by Ian out of a real desire to communicate to young people the relevance of science to their own lives. The value of such an equal partnership and commitment amounts to more than just a combination of expertise and time. Our complementary knowledge and skills helped ensure we optimised all aspects of the lessons. We also benefitted from the detailed planning process required for both of us to understand fully our aims and tactics before entering the classroom. Our goal was to develop a paired approach to make it easier for researchers who are uneasy about working in outreach to commit to engagement. Having two facilitators in the classroom also makes it easier to monitor students’ attention levels, enables dynamic interplay and enables delivery of a synergistic experience.

Working together also pushed us to think through not only what we were going to do, but also exactly how and why to do it, at a level of detail that we may not have reached individually. In our preparatory discussions, we challenged each other about the science and the educational value of each activity. This process inevitably placed us in a stronger position to provide guidance for other facilitators. Such collaboration also has benefits in the form of skills development for science communicator and scientist alike.

Our completed lessons are now freely available online and have also been widely used in schools and in training events. We achieved this dissemination with the support of EuroStemCell, which provided an online platform already used by teachers and researchers, an extensive researcher network for lesson distribution and resources for translation. By 6^th^ November 2014, the lessons had received 7,133 page views from 5,450 unique visitors on eurostemcell.org. Almost 300 print kits of the first lesson in five different languages have also been distributed to educators and scientists throughout the UK and Europe. Translations of all the lessons are underway and will be available to download for free in English, German, French, Spanish, Italian and Polish by February 2015.

In addition, the EuroStemCell team has delivered public engagement training workshops using our lesson materials to 89 researchers from across Europe. Continuing professional development workshops for science teachers from the UK (> 200), Germany (37) and Spain (15) have also taken place using the lessons as an example resource. In July 2013, the Biology teaching specialist at the UK's National STEM Centre selected the lesson materials for listing on the widely used National STEM Centre e-Library for teachers (http://tinyurl.com/oagtpnb). In February 2014, The Guardian's Teacher Network chose to feature the first lesson (http://tinyurl.com/l39uaav) in its article, “How to teach…stem cell research”, which listed our materials as a useful resource for “understanding stem cells”.

We hope that in the next few years, many more colleagues and teachers will find a use for our material and draw on our experience to develop and deliver creative, fun and effective lessons for school children.

